# Peripheral Mechanisms of Dental Pain: The Role of Substance P

**DOI:** 10.1155/2012/951920

**Published:** 2012-02-09

**Authors:** Paola Sacerdote, Luca Levrini

**Affiliations:** ^1^Department of Pharmacology, University of Milan, 20129 Milan, Italy; ^2^Dental Clinic, University of Insubria, Via Piatti 10, Velate, 22100 Varese, Italy

## Abstract

Current evidence supports the central role of neuropeptides in the molecular mechanisms underlying dental pain. In particular, substance P, a neuropeptide produced in neuron cell bodies localised in dorsal root and trigeminal ganglia, contributes to the transmission and maintenance of noxious stimuli and inflammatory processes. The major role of substance P in the onset of dental pain and inflammation is increasingly being recognised. Well-grounded experimental and clinical observations have documented an increase in substance P concentration in patients affected by caries, pulpitis, or granulomas and in those undergoing standard orthodontic or orthodontic/dental care procedures. This paper focuses on the role of substance P in the induction and maintenance of inflammation and dental pain, in order to define future lines of research for the evaluation of therapeutic strategies aimed at modulating the complex effects of this mediator in oral tissues.

## 1. Introduction

Pain is a widely accepted consequence of oral pathological conditions and orthodontic procedures and represents one of the major concerns for both patients and dentists [1]. It is common known that the perception of dental pain is due, at least in part, to an inflammatory reaction that involves different molecular mechanisms. Peripheral pain mechanisms associated with odontogenic painful conditions are overall similar to the mechanisms observed in all other body parts. These similarities include the type of sensory neurons involved as well as the different molecules that play a role in these processes (e.g., receptors, channels, transmitters, and intracellular signalling effectors responsible for the transduction, modulation, and propagation of peripheral stimuli) [2–5]. The pain signal is conducted via thin fibers containing unmyelinated C-fibers and myelinated A-*δ* fibers of primary sensory neurons to secondary order neurons in the spinal cord and finally to the cortex via a relay in the thalamus [3].

Peripheral terminal of nociceptors is environmental detectors that are biochemically specialized by the expression and localization of various receptors and ion channel which confer to these cells the ability to detect noxious chemical, thermal, and mechanical stimuli [3, 4].

A broad range of inflammatory mediators is produced by different cell types in response to tissue injury, and these mediators play different roles in the modulation of pain sensation [3]. The released inflammatory mediators act on the specific receptors expressed in nociceptive sensory neuron and result in the production of second messengers and the downstream activation of protein kinase and phospholipases. The second messengers regulate the activity of the many receptors and ion channels, leading to peripheral sensitization. The ion channels open in response to noxious stimuli initiating and propagating action potentials in the sensory neurons [4]. Neuropeptides are now considered major determinants of the inflammatory process in peripheral tissues, a phenomenon also known as neurogenic inflammation [6]. Mounting evidence is also supporting the role of neuropeptides in the molecular mechanisms underlying dental pain [7].

Substance P (SP) is a neuropeptide produced in a subset of capsaicin sensitive sensory peripheral neuron cell bodies localised in dorsal root and trigeminal ganglia, which plays a pivotal role in the transmission of noxious stimuli in the spinal cord [8]. Moreover, the stimulation of capsaicin-sensitive sensory peripheral terminal of the neurons results in the peripheral release of several neuropeptides, including SP [9, 10].

This brief work focuses on the role of SP in the induction and maintenance of inflammation and dental pain as well as possible future lines of research. We have to remark that other neuropeptides beside SP are present in the same capsaicin sensitive sensory neurons, with either proinflammatory, such as calcitonin gene-related peptide (cGRP) and neurokinin A-B, or antinflammatory activity such as galanin and somatostatin [3–8]. However SP can be considered the more representative peptide involved in neurogenic inflammation and it is the most studied in dental physiology and pathology. Key articles were identified by a targeted MEDLINE search using appropriate keywords (e.g., substance P AND dental pain), which was then manually refined by browsing the reference lists of retrieved articles and by adding other relevant papers according to the authors' knowledge of the field.

## 2. Structure and Mechanisms of SP

SP is an s undecapeptide (H-Arg1-Pro2-Lys3-Pro4-Gln5-Gln6-Phe7-Phe8-Gly9-Leu10-Met11-NH2) and belongs to the same neuropeptide family as neurokinin (NK) A and NK B [9, 11], all of which share the same carboxyl terminal sequence, Phe-X-Gly-Leu-Met-NH2. SP is encoded by the preprotachykinin-A gene in the perikaryon of primary afferent neurons in the dorsal root and trigeminal ganglia and then is transported to both central and peripheral processes of these elements [9, 11]. Interestingly, most (around 80%) of the SP synthesised in dorsal root ganglia is exported towards the terminal regions of their peripheral branches rather than centrally [9–11]. A number of enzymes are involved in the metabolism of substance P, due to their specific cellular localization it is probably Neutral endopeptidase and angiotensin converting enzymes (EP and/or ACE) which are most commonly involved in the cleavage of substance P within the periphery [11].

It is widely accepted that several factors can activate and/or sensitise nociceptors at the site of tissue injury [8, 12] and induce neuropeptide release in the periphery. Capsaicin, heat, and protons activate vanilloid receptor 1 (VR1), which is localized on small diameter sensory fibers resulting in opening of a cation channel, increasing calcium entry through this channel and through voltage-gated calcium channels activated by sodium–induced depolarization. These effects increase SP release from sensory neurons. Bradikinin binds to B2 receptors on sensory neurons and this results in the stimulation of phospholipase C pathway with the activation of the protein kinase C (PKC) cascade that has been demonstrated to stimulate SP secretion from sensory endings. Other compounds do not directly excite sensory neurons but sensitize them, since they lower the threshold for firing. Prostaglandins, produced in inflamed tissue, bind to their receptor on sensory fibers and lower the firing thresholds of neurons throughout cAMP and protein kinase A. As a consequence they also enhance SP release in response to capsaicin, bradikinin, and other stimuli. Finally other mediators such as NGF, that binds to the high affinity TrkA receptors forming an NGf/TrkA complex that is internalized and transported to the neuron cell body, can sensitize the nociceptor [6].

The biological effects of released SP are induced following its binding to specific G protein-coupled NK receptors [11, 13]. There are three types of tachykinin receptors, NK1, NK2, and NK3 exhibiting preferences for substance P, neurokinin A, and neurokinin B, respectively, [11, 13]. However, endogenous tachykinins are not highly selective for any given receptor, and all can act on all three receptors under certain conditions such as receptor availability or at high peptide concentrations.Substance P primarily acts on NK1 receptors and simulation of the NK1 receptor induces several second messengers systems, such as phospholipase C intracellular inositol 1,4,5-trisphophate (IP3) turnover with subsequent elevation of intracellular calcium [11].

These receptors are present in high concentrations in dental tissues [14, 15].

 Moreover SP has been shown to activate ERK 2 and P38 mitogen-activated protein and to increase the production of prostaglandin E2 and the expression of COX2 [16, 17].

**Figure 1:**
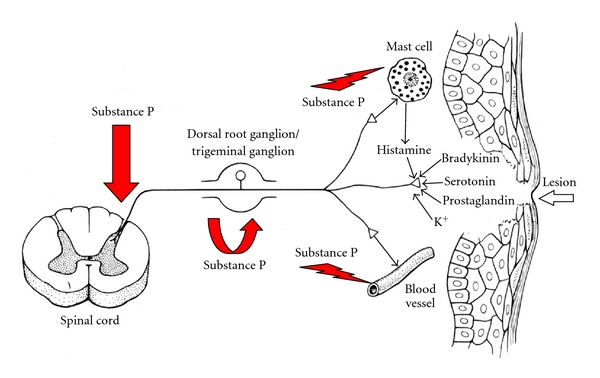
The role of substance P in neurogenic inflammation.

The interaction of SP with its receptors directly induces vasodilatation with increased blood vessel permeability and allows plasma extravasation and mastocyte degranulation. The mastocyte granules release histamine, which in turn further amplifies vascular processes and activates nociceptors [18]. Lymphocytes, granulocytes, and macrophages have receptors for SP and these cells can be stimulated to produce cytokines. Macrophages stimulated by SP produce the inflammatory mediators PGE2, thromboxane, as well as the proinflammatory cytokines IL-1, IL-6, and TNF [11]. All these molecular events ultimately sustain the synthesis and release of new SP, therefore perpetrating the vicious circle (Figure 1). Moreover, these mechanisms do not involve only fibers at the site of tissue damage but are extended also to surrounding undamaged tissues, where they cause secondary hyperalgesia.

On this basis, SP can be considered a major mediator of neurogenic inflammation and associated hyperalgesia and represents a promising target for therapies aimed at controlling pain and minimising deleterious consequences of tissue injury.

## 3. Dental Pulp Innervation and SP

The tooth pulp is a soft connective tissue that is densely innervated and highly vascularised. It is enclosed by rigid mineralised dentin, which strongly limits the ability of the tissue to increase in volume during inflammation and decreases the level of immune defence [2]. Nerve fibers in the pulp include afferent (sensory) fibers originating from the trigeminal ganglia and sympathetic fibers originating from the cervical sympathetic ganglia. Parasympathetic innervation is also present [7].

The number of trigeminal sensory fibers in dental pulp is very high, and several types of sensory fibers are present in this tissue; therefore, the stimulation of pulpal nerves results mainly in pain sensations [2, 19, 20]. For instance, one single human premolar contains 2300 axons at the apex, 87% of which are unmyelinated [21]. Recent findings indicate that the regulation of innervation density is a dynamic process, and the number of nerve fibers can increase in the presence of caries or following orthodontal procedures [2].

SP is abundantly contained in the fibers that innervate the dental pulp and dentin [7]. The production and release of this molecule are highly increased upon noxious, thermal, mechanical, and chemical stimulation of the dental pulp as well as in periodontal ligament [7, 19, 22–24]. The amount of SP released by each sensory fiber is further increased during inflammatory processes, which sustains the vicious circle that underlies inflammation [23, 25]. A number of studies have measured SP concentrations in the human dental pulp, reporting an increase up to 100-fold in inflamed teeth and up to 1000-fold in irreversible pulpitis [25, 26].

## 4. SP and Its Receptors in Dental Pulp

SP exerts several effects in dental pulp (Table 1) [7]. A few studies have characterized the presence of NK receptors in rodent and human teeth [27, 28]. Animal studies identified the expression pattern of the tachykinin receptors NK1, NK2, and NK3 in different types of hard tissue cells, epithelial cells, fibroblasts, endothelium, and blood vessel walls in teeth and supporting oral tissues. NK1 and NK2 receptors have been reported in odontoblast as well as in enamel forming cells (ameloblasts). As expected NK1 receptor is abundantly present on capillaries and smaller blood vessels, and both NK1 and NK2 receptors are densely distributed on the capillary plexus subjacent to dentin [27].

A pronounced density of NK2 receptor has been detected in gingival and Malassez epithelium. NK1 and NK2 receptors were also shown on fibroblasts in the periodontal ligament and in the pulp [27]. Substance P receptors have been reported also in human pulp tissue, although the methods used for their evaluation, radioreceptor assay, did not allow to measure which type of receptor (NK1, NK,2 or NK3) was primarily present [28].

Sensory fibers terminate near blood vessels, and SP receptors are present in high concentrations in this tissue [27, 28]. In healthy tissues, basal SP release plays a key role in the maintenance of tissue homeostasis; on the other hand, massive release of this molecule due to external stimuli induces a vasodilator response followed by a long-lasting increase in blood flow [7]. The increased production and release of SP contributes to the initiation and propagation of the inflammatory process.

SP interacts with mast cells and induces the release of histamine, therefore causing elevated vascular permeability and increased blood pressure [29]. Moreover, lymphocytes, granulocytes, and macrophages contain receptors for SP, and these cells can be stimulated by SP to produce proinflammatory mediators and cytokines [30, 31]. SP also acts as a potent chemotactic agent, which recruits further inflammatory cells in the pulp tissue [30]. The large number of inflammatory and nociceptive mediators dramatically sensitises the nociceptors, stimulates them to release more SP both in the spinal cord and in the dental pulp, and further increases pain sensitivity [7].

## 5. SP in Pathological Dental Conditions

Almost all pathological conditions that affect oral tissues, as well as orthodontic or dental care procedures, increase the production and release of SP (Table 2).

The results of an *ex vivo* study have shown that SP expression is significantly greater in grossly carious painful molars than in grossly carious asymptomatic molars [25]. Mean extracellular levels of SP were >8-fold greater in teeth diagnosed with irreversible pulpitis than in dental pulp diagnosed as normal, as reported by a study in 21 patients, and an increase in SP concentration was observed in granuloma tissues, when compared with healthy controls [26, 32]. Interestingly, also Substance P receptor expression in human pulp tissue is significantly increased during inflammatory phenomena such as acute irreversible pulpitis [28].

Considering that SP exerts its biological activity mainly via the high affinity NK1 receptors but that at higher concentrations the peptide can activate also NK2 and NK3 receptors, it can be speculated that while in physiological condition NK1 is the most involved receptors, in pathological conditions, due to the higher SP concentrations, also NK1 and NK2 become involved.

It has been suggested that SP innervation and SP-related neurogenic inflammation could play a role in several orthodontic procedures.

Deep cavity preparation (<1 mm remaining dentine thickness) for orthodontic reasons was associated with a significant increase in the concentration of SP in pulp tissue, compared with controls [33]. It is well known that root canal preparation generates an inflammatory process in the periapical tissues, which could explain the posttreatment pain events after root canal therapy (such as symptomatic apical periodontitis). Interestingly, it was demonstrated that SP is released in the periodontal ligaments as a result of the application of different canal preparation techniques, although the amount of released SP differs among procedures. SP increase could generate an inflammatory process in the periapical tissues [34, 35].

A recent study has also shown that dentin bonding agents used in restorative dentistry can moderately increase SP release in the dental pulp. This effect is likely to be a result of reactive oxygen species that are formed, which have the ability to diffuse through dentinal tubules, constituting a biological risk to the pulp. Because cavity preparation and the use of dentine bonding agents cooccur very often, a final significant alteration of SP concentrations is likely to occur, that participates in neurogenic inflammation [36].

Similarly, an increase in SP expression and release was observed in patients undergoing orthodontic tooth movement, which was associated with enhanced synthesis of proinflammatory cytokines [34, 37].

Interestingly the kinetic of SP release parallels the perception of pain during orthodontic treatment, which suggests that the perception of pain may be linked to the release of SP [38–40].

Further studies have analyzed the impact of different bracket systems on SP concentrations, with the aim of reducing the inflammation and pain resulting from these procedures [34].

A high level of SP expression has also been reported following procedures with a low impact on tooth physiology, such as light- and laser-activated tooth bleaching [22].

Although it is not possible to reach a conclusion about the biological role of SP release during orthodontic movement, it can be suggested that its release as a consequence of the stimulation of peripheral nerve terminals by means of orthodontic forces may trigger a biochemical cascade which comprises the activation of various types of periodontal ligament cells that could eventually favour inflammatory processes.

## 6. Conclusions and Future Directions

Overall, evidence collected so far strongly supports the major role of SP in the development and maintenance of dental pain and inflammation. An increase in the expression and release of this molecule has been demonstrated following several pathological conditions and common dental procedures. Although physiological levels of SP are needed in order to guarantee correct blood flow in the dental pulp that can help tissue healing, from the available literature it clearly appears that high levels of the neuropeptide are associated with pain and persistent inflammation. Clinicians should be aware that most orthodontic procedures have an impact on the pulpodentine complex, which could generate several neurogenic and vascular reactions, including the release of SP. Although the number of reports in the literatures is only few, there is emerging the evidence that not all orthodontic procedures have a similar impact on SP release [34, 35, 37]. It should therefore become possible to choose approaches with limited effects on SP release.

A second approach to blunt the consequences of SP would be to target with appropriate pharmacological treatment either SP release or SP binding to its receptors present in the pulp. It has been documented that the injection of local anaesthetics results in the attenuation of SP release [19, 41]. This strategy, although potentially useful for limiting the increase in SP concentration, cannot be applied for all conditions and becomes almost useless for the treatment of chronic disease. The use of modulators or blockers of SP receptors has been recently suggested [6], but further evidence is required to fully characterise the benefit/risk ratio of these molecules. Although many studies show the antinociceptive effects of NK1 receptor antagonists, several clinical trials have failed to demonstrate the analgesic efficacy of these compounds in humans [42, 43], though they have been successful in the treatment of other diverse conditions, including depression, chemotherapy-induced emesis, and inflammatory bowel disease [44]. Future studies are necessary to evaluate the usefulness of NK1 receptor antagonists to treat dental pain.

To our knowledge, the effects on SP expression and release of common analgesic treatments used to control pain after dental procedures have not been intensely investigated to date. For instance, nimesulide reduces the concentration of pain mediators, including SP, in patients with knee osteoarthritis [45], but the effects of this agent in dental pain have not been assessed. However, the same pharmacologic effects in dental tissues cannot be excluded and can be extended to other nonsteroidal anti-inflammatory drugs (NSAIDs), although *ad hoc* studies are required to confirm or discard these hypotheses. Therefore, we believe that careful, evidence-based evaluation of the effects of single NSAIDs on the modulation of SP expression, neurogenic inflammation, and sensory neuron excitability in the dental pulp should be pursued in the future.

## Figures and Tables

**Table 1:** Key effects of substance P in dental pulp.

Healthy tissues
Maintenance of tissue homeostasis [6, 27, 28]
Inflamed tissues
Vasodilatatory response [6, 7]
Histamine release [29]
Increase in blood flow [29]
Increase in vascular permeability [29]
Increase in blood pressure [29]
Synthesis of proinflammatory cytokines [30, 31]
Chemotaxis of inflammatory cells [30]
Sensitisation of nociceptors [7]

**Table 2:** Conditions and dental care or orthodontic procedures associated with increased substance P release.

Condition
Caries [25]
Pulpitis [26]
Granuloma [32]

Procedures
Cavity preparation [33]
Tooth movement [34, 35]
Tooth bleaching [22]

## References

[1] Krishnan V (2007). Orthodontic pain: from causes to management—a review. *European journal of orthodontics*.

[2] Henry MA, Hargreaves KM (2007). Peripheral mechanisms of odontogenic pain. *Dental Clinics of North America*.

[3] Coutaux A, Adam F, Willer JC, Le Bars D (2005). Hyperalgesia and allodynia: peripheral mechanisms. *Joint Bone Spine*.

[4] Hucho T, Levine JD (2007). Signaling pathways in sensitization: toward a nociceptor cell biology. *Neuron*.

[5] Gibbs JL, Melnyk JL, Basbaum AI (2011). Differential TRPV1 and TRPV2 channel expression in dental pulp. *Journal of Dental Research*.

[6] Richardson JD, Vasko MR (2002). Cellular mechanisms of neurogenic inflammation. *Journal of Pharmacology and Experimental Therapeutics*.

[7] Caviedes-Bucheli J, Muñoz HR, Azuero-Holguín MM, Ulate E (2008). Neuropeptides in dental pulp: the silent protagonists. *Journal of Endodontics*.

[8] Seybold VS (2009). The role of peptides in central sensitization. *Handbook of Experimental Pharmacology*.

[9] Maggi CA (1995). Tachykinins and calcitonin gene-related peptide (CGRP) as co-transmitters released from peripheral endings of sensory nerves. *Progress in Neurobiology*.

[10] White DM (1997). Release of substance P from peripheral sensory nerve terminals. *Journal of the Peripheral Nervous System*.

[11] Harrison S, Geppetti P (2001). Substance P. *International Journal of Biochemistry and Cell Biology*.

[12] Cheng JK, Ji RR (2008). Intracellular signaling in primary sensory neurons and persistent pain. *Neurochemical Research*.

[13] Snijdelaar DG, Dirksen R, Slappendel R, Crul BJP (2000). Substance P. *European Journal of Pain*.

[14] Fristad I, Vandevska-Radunovic V, Fjeld K, Wimalawansa SJ, Hals Kvinnsland I (2003). NK1, NK2, NK3 and CGRP1 receptors identified in rat oral soft tissues, and in bone and dental hard tissue cells. *Cell and Tissue Research*.

[15] Park CK, Bae JH, Kim HY (2010). Substance P Sensitizes P2X3 in nociceptive trigeminal neurons. *Journal of Dental Research*.

[16] Sun J, Ramnath RD, Zhi L, Tamizhselvi R, Bhatia M (2008). Substance P enhances NF-*κ*B transactivation and chemokine response in murine macrophages via ERK1/2 and p38 MAPK signaling pathways. *American Journal of Physiology*.

[17] Bianchi M, Franchi S, Ferrario P, Sotgiu ML, Sacerdote P (2008). Effects of the bisphosphonate ibandronate on hyperalgesia, substance P, and cytokine levels in a rat model of persistent inflammatory pain. *European Journal of Pain*.

[18] Moriarty D, Selve N, Baird AW, Goldhill J (2001). Potent NK1 antagonism by SR-140333 reduces rat colonic secretory response to immunocyte activation. *American Journal of Physiology*.

[19] Caviedes-Bucheli J, Rojas P, Escalona M (2009). The effect of different vasoconstrictors and local anesthetic solutions on substance P expression in human dental pulp. *Journal of Endodontics*.

[20] Fried K, Lillesaar C, Sime W, Kaukua N, Patarroyo M (2007). Target finding of pain nerve fibers: neural growth mechanisms in the tooth pulp. *Physiology and Behavior*.

[21] Walton RE, Ramachandran Nair PN (1995). Neural elements in dental pulp and dentin. *Oral Surgery, Oral Medicine, Oral Pathology, Oral Radiology and*.

[22] Caviedes-Bucheli J, Ariza-García G, Restrepo-Méndez S, Ríos-Osorio N, Lombana N, Muñoz HR (2008). The effect of tooth bleaching on substance P expression in human dental pulp. *Journal of Endodontics*.

[23] Caviedes-Bucheli J, Azuero-Holguin MM, Correa-Ortiz JA (2011). Effect of experimentally induced occlusal trauma on substance P expression in human dental pulp and periodontal ligament. *Journal of Endodontics*.

[24] Awawdeh LA, Lundy FT, Linden GJ, Shaw C, Kennedy JG, Lamey PJ (2002). Quantitative analysis of substance P, neurokinin A and calcitonin gene-related peptide in gingival crevicular fluid associated with painful human teeth. *European Journal of Oral Sciences*.

[25] Rodd HD, Boissonade FM (2000). Substance P expression in human tooth pulp in relation to caries and pain experience. *European Journal of Oral Sciences*.

[26] Bowles WR, Withrow JC, Lepinski AM, Hargreaves KM (2003). Tissue levels of immunoreactive substance P are increased in patients with irreversible pulpitis. *Journal of Endodontics*.

[27] Kido MA, Ibuki T, Danjo A (2005). Immunocytochemical localization of the neurokinin 1 receptor in rat dental pulp. *Archives of Histology and Cytology*.

[28] Caviedes-Bucheli J, Gutierrez-Guerra JE, Salazar F, Pichardo D, Moreno GC, Munoz HR (2007). Substance P receptor expression in healthy and inflamed human pulp tissue. *International Endodontic Journal*.

[29] Györfi A, Fazekas A, Irmes F, Rosivall L (1995). Effect of substance P administration on vascular permeability in the rat oral mucosa and sublingual gland. *Journal of Periodontal Research*.

[30] Delgado AV, McManus AT, Chambers JP (2003). Production of tumor necrosis factor-alpha, interleukin 1-beta, interleukin 2, and interleukin 6 by rat leukocyte subpopulations after exposure to substance P. *Neuropeptides*.

[31] Park SH, Hsiao GYW, Huang GTJ (2004). Role of substance P and calcitonin gene-related peptide in the regulation of interleukin-8 and monocyte chemotactic protein-1 expression in human dental pulp. *International Endodontic Journal*.

[32] Kabashima H, Nagata K, Maeda K, Iijima T (2002). Involvement of substance P, mast cells, TNF-*α* and ICAM-1 in the infiltration of inflammatory cells in human periapical granulomas. *Journal of Oral Pathology and Medicine*.

[33] Caviedes-Bucheli J, Correa-Ortíz JA, García LV, López-Torres R, Lombana N, Muñoz HR (2005). The effect of cavity preparation on substance P expression in human dental pulp. *Journal of Endodontics*.

[34] Yamaguchi M, Takizawa T, Nakajima R, Imamura R, Kasai K (2009). The damon system and release of substance p in gingival crevicular fluid during orthodontic tooth movement in adults. *World Journal of Orthodontics*.

[35] Caviedes-Bucheli J, Azuero-Holguin MM, Gutierrez-Sanchez L (2010). The effect of three different rotary instrumentation systems on substance p and calcitonin gene-related peptide expression in human periodontal ligament. *Journal of Endodontics*.

[36] Caviedes-Bucheli J, Correa-Ortiz JA, Ballestero AC (2010). The effect of dentine-bonding agents on substance P release in human dental pulp. *International Endodontic Journal*.

[37] Kojima T, Yamaguchi M, Kasai K (2006). Substance P stimulates release of RANKL via COX-2 expression in human dental pulp cells. *Inflammation Research*.

[38] Yamaguchi M, Yoshii M, Kasai K (2006). Relationship between substance P and interleukin-1*β* in gingival crevicular fluid during orthodontic tooth movement in adults. *European Journal of Orthodontics*.

[39] Ertan Erdinç AM, Dinçer B (2004). Perception of pain during orthodontic treatment with fixed appliances. *European Journal of Orthodontics*.

[40] Feldmann I, List T, Bondemark L (2012). Orthodontic anchoring techniques and its influence on pain, discomfort, and jaw function—a randomized controlled trial. *Journal of Orthodontics*.

[41] Pertl C, Amann R, Odell E, Robinson PD, Kim S (1997). Effects of local anesthesia on substance P and CGRP content of the human dental pulp. *Journal of Endodontics*.

[42] Hill RG, Oliver KR (2007). Neuropeptide and kinin antagonists. *Handbook of Experimental Pharmacology*.

[43] Duffy RA (2004). Potential therapeutic targets for neurokinin-1 receptor antagonists. *Expert Opinion on Emerging Drugs*.

[44] Ambalavanar R, Dessem D (2009). Emerging peripheral receptor targets for deep-tissue craniofacial pain therapies. *Journal of Dental Research*.

[45] Bianchi M, Broggini M, Balzarini P, Franchi S, Sacerdote P (2007). Effects of nimesulide on pain and on synovial fluid concentrations of substance P, interleukin-6 and interleukin-8 in patients with knee osteoarthritis: comparison with celecoxib. *International Journal of Clinical Practice*.

